# Assessing the Influence of Different ROI Selection Strategies on Functional Connectivity Analyses of fMRI Data Acquired During Steady-State Conditions

**DOI:** 10.1371/journal.pone.0014788

**Published:** 2011-04-13

**Authors:** Guillaume Marrelec, Peter Fransson

**Affiliations:** 1 U678, Inserm, Paris, France; 2 UMR-S 678, Université Pierre et Marie Curie Paris 6, Paris, France; 3 LINeM, Inserm, Université de Montréal Montreal, Canada, and Université Pierre et Marie Curie Paris 6, Paris, France; 4 Department of Clinical Neuroscience, Karolinska Institute, Stockholm, Sweden; University of Illinois at Chicago, United States of America

## Abstract

In blood oxygen level dependent (BOLD) functional magnetic resonance imaging (fMRI), assessing functional connectivity between and within brain networks from datasets acquired during steady-state conditions has become increasingly common. However, in contrast to connectivity analyses based on task-evoked signal changes, selecting the optimal spatial location of the regions of interest (ROIs) whose timecourses will be extracted and used in subsequent analyses is not straightforward. Moreover, it is also unknown how different choices of the precise anatomical locations within given brain regions influence the estimates of functional connectivity under steady-state conditions. The objective of the present study was to assess the variability in estimates of functional connectivity induced by different anatomical choices of ROI locations for a given brain network. We here targeted the default mode network (DMN) sampled during both resting-state and a continuous verbal 2-back working memory task to compare four different methods to extract ROIs in terms of ROI features (spatial overlap, spatial functional heterogeneity), signal features (signal distribution, mean, variance, correlation) as well as strength of functional connectivity as a function of condition. We show that, while different ROI selection methods produced quantitatively different results, all tested ROI selection methods agreed on the final conclusion that functional connectivity within the DMN decreased during the continuous working memory task compared to rest.

## Introduction

Since the seminal work of Biswal et al. [1], there has been a steady increase in the interest to investigate steady-state activity in networks that are driven by spontaneous, intrinsic MR signal intensity fluctuations. Correlation-based functional connectivity, which refers to the statistical covariations of the blood oxygen level dependent (BOLD) signal in different parts of the brain [2], is a common way to use functional magnetic resonance imaging (fMRI) data to this end. An important step of many functional connectivity analyses consists of selecting representative spatial locations, or regions of interest (ROIs), from which signal intensity time courses will be extracted. In the case of functional connectivity analyses performed on task-evoked data, this procedure is often facilitated by the fact that the investigator's choice is guided by either the spatial locations that show the largest activations and/or deactivations in response to the given task within a certain brain area or, alternatively, by information obtained from previous studies.

However, selecting anatomical locations within ROIs for functional connectivity analyses performed on data acquired during steady-state conditions is often less straightforward (see, e.g., recent review [3] and [4]). The primary reason for this is that there is often no or little prior information regarding the optimal anatomical location of the ROIs that should reflect intrinsic activity in any given brain area. For example, in case of functional connectivity studies of the default mode network (DMN), previous investigations have used data from independent task-evoked studies to locate suitable locations for ROIs, which might or might not constitute an optimal choice to investigate functional connectivity of low-frequency, spontaneous signal fluctuations in the DMN [5]–[7]. Assessing the validity of methods for fMRI data analysis is a key issue but, for lack of gold standard, also a thorny one. Still, some efforts have been made to assess the validity of the methods used. Some studies have tried to assess the effect of preprocessing on the data and its robustness to certain parameters [8], [9]; a general framework to evaluate preprocessing was also proposed [10]. Regarding functional connectivity, we are aware of only few attempts. Himbert et al. [11] and Damoiseaux et al. [12] investigated the reproducibility and robustness of spatial independent component analysis (sICA) . While Vincent et al. [13] found that correlation maps in monkeys were robust to the choice of the seed region when it is located within the oculomotor system, Margulies et al. [14] showed that even a small shift of the seed voxel within the precuneus could lead to significant changes of the connectivity pattern; similar results were reported using slightly different ROIs within the DMN [3]. Since functional connectivity estimated from intrinsic BOLD activity is routinely used to characterize differences in networks in patient populations (see, e.g., [15]–[17]), we sought to investigate the impact that different ROI selection methods might have on the resulting connectivity measures. More specifically, our objective was to answer the following question: Given a specific set of ROIs (i.e., brain regions), how similar are the volumes and time courses extracted by different ROI selection methods, and how similar are results from connectivity analyses performed on these volumes and time courses? Indeed, since the results of [14] and [3], one could wonder if different ROI selection methods that aim at extracting a specific set of ROIs provide consistent results. For the present study, we focused on the relationship between different choices of ROI selection methods and functional connectivity within the DMN during both continuous rest and a verbal 2-back working memory task [18]. Four different strategies of ROI selection were compared: (a) ROIs centered on the coordinates given in [6] (TalFox), (b) ROIs centered on the coordinates provided in [7] (TalFr), (c) ROIs centers obtained from a group-level independent component analysis (gICA) of the dataset, and (d) selection of ROIs based on independent component analyses performed at the individual level (indICAs). Note that, while the anatomical locations of the ROIs were the same across subjects in schemes a–c, the exact centers of the ROIs were allowed to vary from subject to subject in scheme (d).

We compared the four different ROI selection strategies at three consecutive steps of the analysis. In Step 1, we examined some spatial and functional features of the regions extracted by the four ROI selection methods. In Step 2, we compared the signals extracted by the four methods in terms of temporal distribution, mean and variance. Steps 1 and 2 are general, in that they compared general features of the signals regardless of their subsequent use. In Step 3, we considered the effect that the four ROI selection methods had on a functional connectivity analysis. In this step, we examined to which extent changes in features induced by different ROI selection approaches had an influence on the results of functional connectivity. Obviously, this step is specific to functional connectivity. While our primary objective was to compare the results provided by the ROI selection methods at this third step, we expected the four methods to produce quantitatively different results and, as a consequence, designed Steps 1 and 2 to better understand at what level and in what measure these methods differed. Furthermore, since knowing that different methods lead to quantitatively different results provides no information as to the confidence that one can have regarding the qualitative interpretation of the results of these methods, we also compared the conclusions that we could draw using each method.

## Analysis

### Data and ROI selection

For the purpose of the present article, we re-analyzed data already published [18], [19].

#### Subjects and tasks

Seventeen subjects (5 males, age span 22–41 years) participated in this study. No subject had any history of neurological or psychiatric illness. All MR examinations were carried out according to the ethical guidelines and declarations of the Declaration of Helsinki (1975) and the current study was approved at the Karolinska University Hospital by the Regionala etikproevningsnaemnden i Stockholm (“the regional ethical committee in Stockholm”). Written consent was obtained from all subjects. All subjects participated in two 10 min echo-planar imaging (EPI) blood oxygenation level dependent (BOLD) scanning sessions during which they either performed a resting-state task with their eyes fixating on a hair-cross centered on a white black screen or engaged in a continuous verbal 2-back working memory task.

#### MR image acquisition

All MRI data was acquired on a General Electric Twin-Speed Signa Horizon 1.5 T MRI scanner. Echo-planar imaging (TR/TE
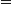
2000/40 ms, flip
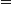
80 degrees, 64
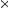
64 matrix size, FOV
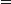
220
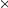
220 mm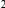, 29 slices) was used to detect BOLD fMRI signal changes during rest and the working memory task. 300 echo-planar image volumes were acquired for each task.

#### Image preprocessing

All image processing was performed using the SPM2 software package (Wellcome Dept. of Imaging Neuroscience, London, UK). As a first step, functional image time-series were corrected for head motion by realigning all images to the first image volume. Second, the mean EPI image for each subject was co-registered to a corresponding 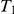-weighted high-resolution image volume and subsequently spatially normalized and re-sampled (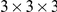 mm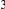 voxels) to the approximate Talairach space [20] as defined by the MNI (Montreal Neurological Institute) EPI template in SPM2. As a last step, the normalized echo-planar image volumes were spatially filtered using an isotropic Gaussian filter (6 mm FWHM).

#### Selecting regions of interest

According to previous studies, nine regions of interest (ROIs) belonging to the default mode network were selected: precuneus/posterior cingulate cortex (pC/pCC), left and right inferior parietal lobes (lIPL, rIPL), dorsal and ventral medial prefrontal cortices (dmPFC, vmPFC), left and right temporal cortices (lTC, rTC) and left and right medial temporal lobes (lMTL, rMTL). All nine ROIs were independently selected following four distinct methods:

- using Talairach coordinates as given in [7], denoted TalFr;
- using Talairach corrdinates as given in [6], denoted TalFox;
- performing group spatial independent component analysis (ICA), denoted gICA; and
- performing individual sICAs, denoted indICAs.

Since the ROI coordinates given in [6] were supplied in Talairach space, we used the nonlinear conversion routine tal2mni.m (http://imaging.mrc-cbu.cam.ac.uk/imaging/MniTalairach) to convert the coordinates to the space defined by the MNI atlas in SPM. ROI selection based on individual ICAs was accomplished as in [19], that is, in a three-step procedure. First, an independent component analysis (ICA) of the resting-state data was performed and 60 spatio-temporal independent components were extracted from each individual dataset using the MELODIC FSL software (MELODIC v4.0; FMRIB Oxford University, UK). Second, by matching each independent component with a spatial template of the default mode network based on an independent dataset [7], the spatially best-fitting independent component was extracted for each subject as previously described [21], [22]. Third, local estimates of default mode activity in each network region were identified in terms of voxels exhibiting local Z-score maxima in the best-fitting independent component. To ensure that only the relevant anatomical structures were included, the search for each local maximum was constrained by using the WFU (Wake Forest University) Pickatlas toolbox [23] together with the AAL (Automatic Anatomical Labelling) atlas [24] within SPM. Consequently, the exact spatial location for the ROIs was allowed to vary between individuals, although only within specified anatomical boundaries [19]. Although the anatomical constraints were set to be rather liberal, the possibility that they impose a user-introduced bias in the ROI selection process can not be fully ruled out. An additional constraint was that the individual regions had to be located at least 15 mm apart. Since the distance between the dorsal and ventral medial prefrontal cortices was less than 15 mm in three subjects, these three subjects were discarded and the subsequent partial and marginal correlation analysis was based on the remaining fourteen subjects.

The group independent component analysis was performed using the temporal concatenation approach to tensorial version of the independent component analysis module implemented in MELODIC.

**Figure 1:**
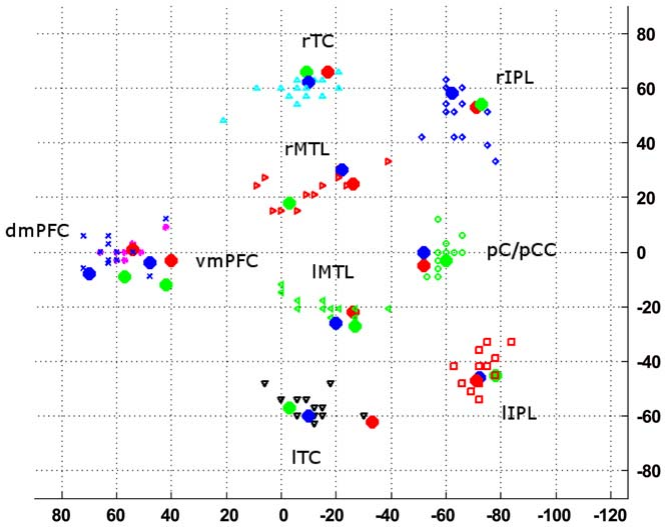
Location of the ROIs used in the present analysis. Large blue filled dots: centers for TalFr; large red filled dots: centers for TalFox; large green filled dots: centers for gICA; green circles: pC/PCC; red squares: lIPL; blue diamonds: rIPL; blue crosses: dmPFC; magenta astrixes: vmPFC; black down-pointing triangles: lTC; cyan up-pointing triangles: rTC; green left-pointing triangles: lMTL; red right-pointing triangles: rMTL.

For a graphical presentation of the location of the ROIs for all four methods, see Figure 1. Regardless of the ROI selection approach, signal intensity time-courses during both rest and the working memory task were extracted using spherical ROIs with a radius of 6 mm. All signal intensity time-courses were bandpass filtered (passband 0.012–0.1 Hz) and orthogonalized with respect to the global mean brain signal. Thus, in each individual, 9 (network regions/nodes)
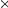
2 (conditions: rest and working memory) BOLD signal intensity time-courses were extracted, resulting in two datasets per subject pertaining to default mode network activity in the nine network regions.

We finally obtained 4 (ROI selection methods)
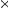
2 (conditions)
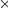
9 (regions)
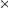
14 (subjects) time series to assess the variability induced by ROI selection. All computations were performed using Matlab (The MathWorks, Inc.).

## Methods

We here introduce several tools that we used for data analysis, namely a measure of within-ROI spatial functional heterogeneity, a test to measure the discrepancy between two probability distribution functions, a series of appproximate nonparametric permutation tests based on 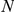-way ANOVA to check for the presence of effects, and, finally, a measure making it possible to quantify the similarity between correlation matrices.

### Assessing within-ROI spatial functional heterogeneity

To quantify the functional heterogeneity of a ROI composed of 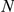 voxels, we defined spatial functional heterogeneity based on the similarity of the time courses of the 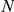 voxels composing the region as follows. We first computed the 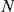-by-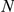 covariance matrix of the 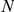 time courses as well as the 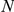 corresponding eigenvalues. If the sum of all eigenvalues were divided randomly between the various components, then the expected distribution of the eigenvalues would follow a broken-stick distribution; observed eigenvalues 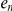 were then kept if they exceeded eigenvalues 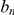 generated by the broken-stick model, i.e. [25], [26]

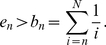
(1)To avoid spurious effects due to the discrete nature of this measure, we defined spatial functional heterogeneity 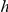 as the (real) value for which the plots of 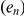 and 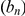 last intersect. If 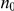 was the last eigenvalue for which we have 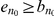 and 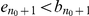, then 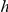 was defined as
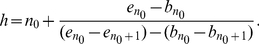
(2)


### Comparing two distributions

Probability distributions were compared using the 2-sample Cramér-von Mises-Smirnov test [27]–[30]. More specifically, let 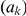 and 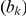 be two samples of size 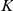 obtained according to two probability distributions 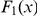 and 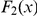, respectively. We test the equality of 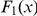 and 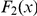 through the quantity
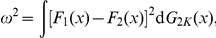
where 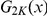 is the distribution obtained by assuming that both samples originate from the same distribution. Let 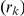 be the ranks of the 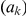 in the combined sample and 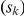 be the ranks of the 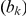 in the combined sample. Then the statistic is
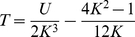
(3)with
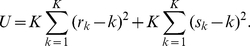
(4)Based on the assumption of large 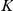, significance levels of the statistic were obtained from [30].

### Testing for normality

In a similar fashion, the hypothesis that a distribution is normal can be tested using the one-sample Cramér-von Mises test, whose goal is to provide an approximation of
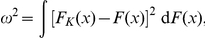
where 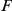 is the theoretical distribution (here, a normal distribution), and 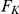 the empirical distribution. Let 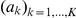 be the time series sorted in increasing order. Then
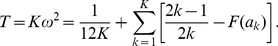
(5)Akin to the 2-sample Cramér-von-Mises-Smirnov test, this statistic was thresholded using tables from [30] and the assumption of large datasets.

### Checking the presence of effects

To ascertain the presence of a global effect of method on the data, we used appproximate nonparametric permutation tests based on 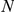-way ANOVA with one replicate. Specifically, denote by 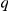 the quantity of interest. Since, in the following, 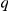 always depends on at least methode 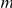, condition 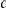 and subject 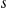, we write 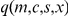, where 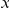 stands for any other set of variables. For instance, in the case of region-dependent scalar measures (e.g., mean, variance), we have 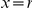 with 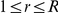. If 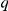 is a global scalar measure (e.g., integration), we have 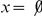. If 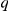 is a global multidimensional measure of dimension 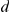 (e.g., correlation matrix: 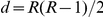; MDS components: 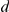 is the number of components), then 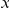 is an index varying from 1 to 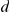. Using standard 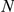-way ANOVA with one replicate [31], we first computed 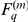, the 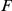 statistic corresponding to an effect of method on 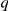. 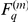 was then transformed into a 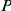-value using the empirical distribution of 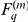 under the null hypothesis as obtained by approximate permutation test [32], [33]. More precisely, we defined a null hypothesis 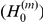 of no effect of method on 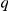. Under 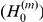, all methods are equivalent. Consequently, for a given condition 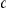, subject 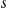 and other variables 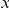, methods are exchangeable and can be randomly permutated, leading to a new set of measures 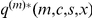, 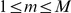 (here 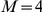) for each random permutation. When 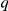 was multidimensional, we preserved its structure by performing the same random permutation to the whole multidimensional structure; for instance, in the case of correlation, all elements of the matrix corresponding to condition 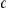 and subject 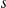 were subject to the same random permutation. Applying 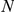-way ANOVA to this synthetic dataset yielded a new statistic 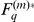. This step was repeated 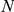 times (here 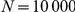), leading to a set of 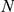 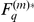 values which were then used as an approximation for the distributions of 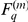 under 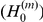. Last, the 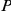-value corresponding to an effect of method in the original dataset was approximated by the fraction of 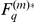 that were above 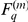.

Pairwise comparison of methods was performed likewise, but for the fact that we each time only considered the two methods under investigation instead of the full set of 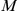 methods. A similar argument was applied to assess the significance of an effect of condition.

Lastly, to validate the consistency of the effects detected across methods and conditions, we also performed 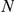-was ANOVAs to test for the presence of a method-specific effect of condition as well as a condition-specific effect of method.

### Comparing correlation matrices

To compare matrices to one another, we resorted to the following metric. For a group of 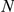 datasets, each dataset 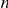 having a correlation matrix 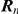, we computed the average correlation matrix 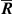 as
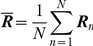
and the corresponding measure of nonhomogeneity, or variability, of that dataset as
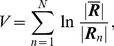
(6)where 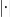 stands for matrix determinant. 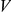 is a normalized version of the minimum discriminant statistic introduced by [34], pp. 318–324, with datasets of equal lengths. It can be shown that 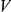 is always positive, with equality if and only if all correlation matrices are equal. As a consequence, 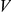 quantified the variability in terms of functional connectivity that can be observed within the 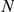 datasets.

### Step 1: ROI features

For each ROI, we first compared the different volumes extracted by the four ROI selection procedures in terms of spatial localization and the temporal heterogeneity of the BOLD signals extracted for all voxels inside the ROIs.

#### Spatial localization

**Figure 2:**
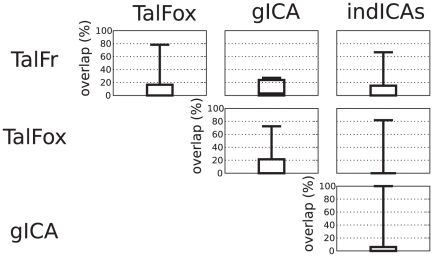
Spatial overlap between ROIs according to the four ROI selection methods. If 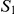 and 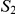 are the spheres extracted for a given ROI by methods 1 and 2, respectively, then the overlap between methods 1 and 2 for that ROI is computed as 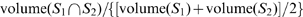. The bottom and top of the box are the 25th and 75th percentile (the lower and upper quartiles, respectively), and the band in the box is the 50th percentile (median); whiskers represent minimum and maximum values.

**Figure 3:**
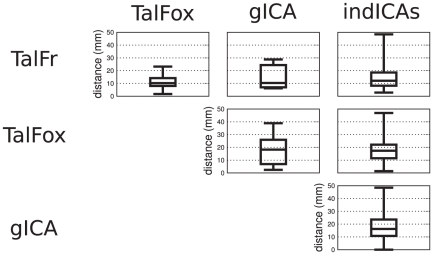
Distance between ROI centers according to the four ROI selection methods. The bottom and top of the box are the 25th and 75th percentile (the lower and upper quartiles, respectively), and the band in the box is the 50th percentile (median); whiskers represent minimum and maximum values.

The first step was to examine the degree of spatial overlap for the same ROI as extracted by different methods. The results are summarized in Figure 2 (for detailed results, see Figures S1 and S2). Since the degree of spatial overlap was equal to zero in many cases, we also reported in Figure 3 the distances between the different peaks extracted by the four methods (for detailed results, see Figures S3 and S4).

#### Functional heterogeneity

**Figure 4:**
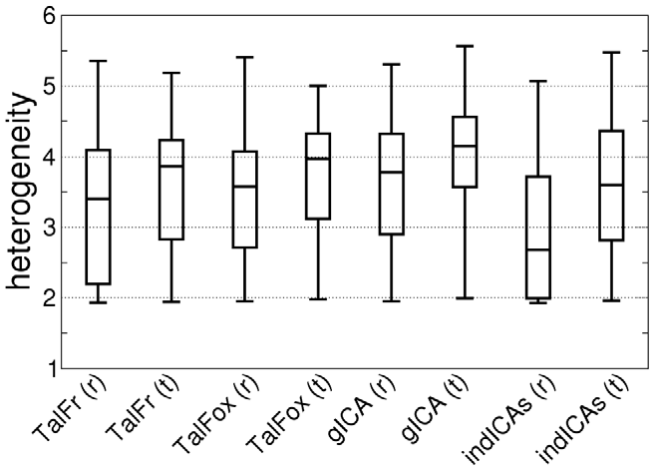
Within-ROI spatial functional heterogeneity as a function of method and condition. The bottom and top of the box are the 25th and 75th percentile (the lower and upper quartiles, respectively), and the band in the box is the 50th percentile (median); whiskers represent minimum and maximum values.

For each ROI extracted, we also investigated the signal of its constituting voxels as follows. We first determined the spatial functional heterogeneity of each ROI using the broken-stick model mentioned in the Methods Section, see Equation (1). Results are summarized in Figure 4. We then examined the potential effect of method and condition. We found an effect for method and condition (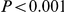 in both cases). All pairwise comparisons between methods are reported in Table 1. As to condition, the effect was an increase in within-ROI spatial functional heterogeneity from rest to the working memory task.

**Table 1:** Pairwise effects of method on within-ROI spatial functional heterogeneity.

(A)	TalFr	TalFox	gICA	indICAs
TalFr	—	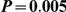	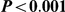	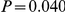
TalFox	TalFox 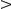 TalFr	—	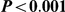	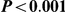
gICA	gICA 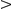 TalFr	gICA 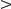 TalFox	—	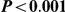
indICAs	n.s.	indICAs 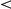 TalFox	indICAs 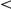 gICA	—

Upper triangular matrix: significance level of an effect of method. Significant 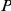-values at a threshold of 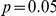 corrected are emphasized in bold. Lower triangular matrix: direction of effect.

**Figure 5:**
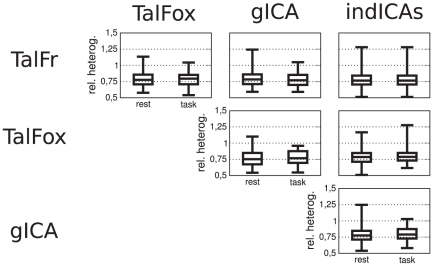
Relative spatial functional heterogeneity as a function of pair of methods and condition. The bottom and top of the box are the 25th and 75th percentile (the lower and upper quartiles, respectively), and the band in the box is the 50th percentile (median); whiskers represent minimum and maximum values.

We also examined the between-method functional similarity of ROIs as follows. Denote by 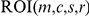 the ROI extracted by method 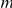 from data corresponding to subject 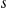 for region 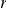 during condition 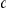, and 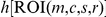 its spatial functional heterogeneity. For each pair of methods 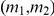, condition 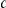, subject 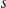, and region 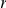, we computed the relative functional heterogeneity as
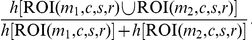
A relative heterogeneity close to 0.5 indicates that 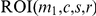 and 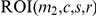 have very similar functional content, while a relative heterogeneity around 1 indicates two ROIs that have voxels with rather distinct time series. We found an effect of method on relative heterogeneity that was barely above the significance level (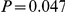) and no effect of condition (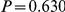). The results are summarized in Figure 5.

### Step 2: General signal features

Methods for functional connectivity analysis usually require one BOLD signal intensity time courses per ROI. This time course is usually obtained as a spatial average of the time courses of all voxels within the ROI. The second step was therefore to assess the effect of method and condition on the sampling distribution of these time courses.

#### Signal distributions

We performed an analysis of the marginal features of the average signal within each ROI by assessing such characteristics as the global shape of the distribution as well as its mean and variance.

#### Signal distributions: whole distributions

We used the approach detailed in the Methods section, see Equations (3) and (4). When examining the effect of method, we had 2 (conditions)
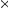
14 (subjects)
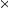
9 (regions)
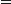
252 tests for each pair of methods 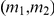 to compare. We found many significant differences (between 61 and 105 at 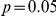 uncorrected, depending on the pair of methods compared; between 33 and 75 at 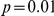 uncorrected; between 17 and 53 at 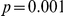 uncorrected). However, most of these differences vanished when ROI signals were scaled to zero mean and unit variance: at 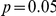 uncorrected, we found only two significant differences and both differences even vanished when the threshold was lowered to 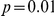 uncorrected. Regarding the effect of condition, we performed 4 (methods)
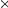
14 (subjects)
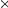
9 (regions)
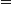
504 tests. We found the same pattern as for the effect of method, namely many significant differences between raw signals (187 at 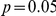 uncorrected; 111 at 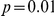 uncorrected; 50 at 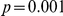 uncorrected), differences that disappeared when the scaled data were considered (3 at 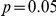 uncorrected; 1 at 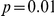 uncorrected; none at 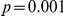 uncorrected).

#### Signal distributions: normality of data

We tested the hypothesis that the signal sampling distributions could be normal using the approach detailed in the Methods section, see Equation (4). We found that, out of the 4 (methods)
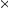
2 (tasks)
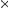
14 (subjects)
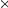
9 (regions)
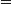
1008 tests performed, very few were significant (15 at 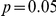 , uncorrected; 5 at 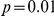, uncorrected; 1 at 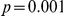, uncorrected).

#### Signal distributions: signal means

Using the approach described in the Methods section, we investigated the influence of method and condition on the ROI signal means. We found no significant effect of method (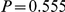) nor condition (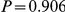).

#### Signal distributions: signal variances

Examining the temporal variances of the representative ROI signals, we found significant effect of both method and condition (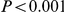 in both cases). All between-method pairwise comparisons are reported in Table 2A. Variance for gICA and indICAs were found to be lower and higher, respectively, than for any other method. By contrast, TalFr and TalFox did not significantly differ. Regarding condition, we found a decrease of variance from rest to task. Regarding this change, we found a significant effect of method on its absolute value (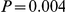) but not on its relative value (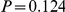). Pairwise comparisons of the between-condition changes are reported in Figure 2B.

**Table 2:** Pairwise effects of method on signal variance.

(A)	TalFr	TalFox	gICA	indICAs
TalFr	—	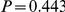	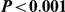	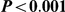
TalFox	n.s.	—	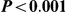	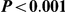
gICA	gICA 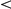 TalFr	gICA 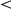 TalFox	—	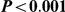
indICAs	indICAs 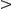 TalFr	indICAs 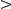 TalFox	indICAs 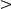 gICA	—
**(B)**				
TalFr	—	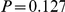	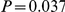	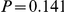
TalFox	n.s.	—	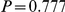	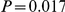
gICA	n.s.	n.s.	—	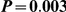
indICAs	n.s.	n.s.	indICAs 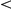 gICA	—

(A) Variance. (B) Absolute variance change. Upper triangular matrix: significance level of an effect of method. Significant 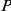-values at a threshold of 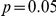 corected are emphasized in bold. Lower triangular matrix: direction of effect; n.s.: nonsignificant. Note that, since changes are usually negative, a larger change means a change that is smaller in amplitude.

#### Between-method regional correlations

Pairwise correlations between two signals observed in the same ROI but obtained with two different methods were also examined. We found that there globally existed a strong correlation between signals extracted from the same region and condition but with different methods. We found a significant effect for both the pair of methods considered and condition (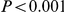 in both cases). No specific pattern was observed for method. For condition, the effect was a decrease when going from continuous rest to the working memory task.

### Differences in patterns of functional connectivity

As a second evaluation step, we investigated the influence of method and condition on functional connectivity and, more specifically, on one global measure (integration), two pairwise measures (marginal and partial correlation), as well as on the global pattern of correlation.

#### Integration

In this first approach, we summarized the information contained in the correlation matrices by computing their integration. Integration is a measure known in information theory and multivariate analysis as total correlation [35], multivariate constraint [36], or multiinformation [37], [38]. In neuroscience, it was first applied to neurocomputing [39]; more recently, it was also applied to functional MRI data analysis [40], [41]. We found an effect of both method and condition (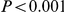 in both cases). With respect to methods, the results of all pairwise comparisons are summarized in Table 3A. Similarly to what was found for variance, integration in gICA and indICAs was found to be lower and larger, respectively, than for TalFr and TalFox. As to the global effect of condition, integration within the DMN during the working memory task was found to be lower than during rest. When considering the change in integration from rest to task, we found a significant effect of method (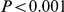) on the the absolute intensity. All pairwise comparisons are reported in Table 3B; effects were only found between indICAs on the one hand and other methods on the other hand, with a larger decrease of the former compared to the latter. We also found a significant effect of method on the relative change in integration (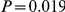), but none of the pairwise comparisons we made exhibited significant differences with a threshold of 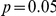 corrected (see Table 3C).

**Table 3:** Pairwise effects of method on network integration.

(A)	TalFr	TalFox	gICA	indICAs
TalFr	—	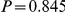	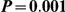	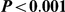
TalFox	n.s.	—	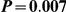	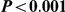
gICA	gICA 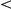 TalFr	gICA 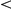 TalFox	—	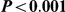
indICAs	indICAs 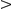 TalFr	indICAs 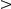 TalFox	indICAs 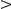 gICA	—
**(B)**				
TalFr	—	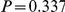	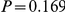	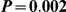
TalFox	n.s.	—	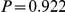	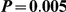
gICA	n.s.	n.s.	—	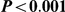
indICAs	indICAs 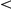 TalFr	indICAs 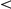 TalFox	indICAs 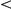 gICA	—
**(C)**				
TalFr	—	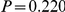	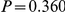	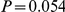
TalFox	n.s.	—	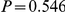	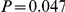
gICA	n.s.	n.s.	—	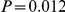
indICAs	n.s.	n.s.	n.s.	—

(A) Integration. (B) Absolute integration change. (C) Relative integration change. Upper triangular matrix: significance level of an effect of method. Significant 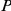-values at a threshold of 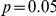 corected are emphasized in bold. Lower triangular matrix: direction of effect; n.s.: nonsignificant. Note that, since changes are usually negative, a larger change means a change that is smaller in amplitude.

#### Marginal correlation

We here examined the effect of method and condition on the marginal correlation coefficients. Correlation has been used as a measure of functional connectivity since the first studies [1], [2], [42]. We found a significant effect for both method and condition (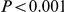 in both cases). All pairwise comparisons are summarized in Table 4A. Globally, IndICAs provided correlation values that were larger than for any other method, gICA with values that tended to be lower. As to condition, its effect was a decrease from rest to task. When we examined the effect of method on this decrease, we found a significant effect of method (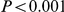) on absolute variation. All pairwise comparisons are reported in Table 4B. The decrease is larger for indICAs than for any other method; it is similar for TalFr, TalFox, and gICA. By contrast, there was no effect of method on the relative variations (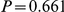).

**Table 4:** Pairwise effects of method on interregional correlation.

(A)	TalFr	TalFox	gICA	indICAs
TalFr	—	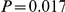	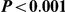	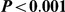
TalFox	n.s.	—	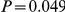	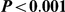
gICA	gICA 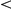 TalFr	n.s.	—	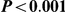
indIAs	indICAs 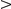 TalFr	indICAs 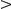 TalFox	indICAs 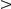 gICA	—
**(B)**				
TalFr	—	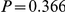	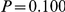	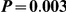
TalFox	n.s.	—	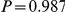	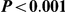
gICA	n.s.	n.s.	—	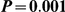
indIAs	indICAs 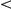 TalFr	indICAs 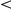 TalFox	indICAs 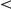 gICA	—

(A) Marginal correlation. (B) Absolute change in marginal correlation. Upper triangular matrix: significance level of a pairwise effect of method. Significant 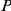-values at a threshold of 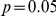 corrected are emphasized in bold. Lower triangular matrix: direction of effect; n.s.: nonsignificant. Note that, since changes are usually negative, a larger change means a change that is smaller in amplitude.

#### Partial correlation

We also examined the effect of method and condition on the partial correlation coefficients. Partial correlation coefficients are here computed as the correlations between any two regions after the effect of the seven other regions onto these two regions have been removed by conditioning. It was used as a measure of functional connectivity that could be closer to effective connectivity than classical (marginal) correlation coefficients [43]–[50]. We found a significant effect for both method and condition (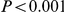 in both cases). Pairwise comparisons can be found in Table 5. Partial correlations were largest for indICAs, lowest for gICA. The effect of condition was a decrease of partial correlation coefficients from rest to task. Regarding the change in partial correlation, we found no significant effect of method for either the absolute change (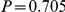) nor the relative change (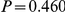).

**Table 5:** Pairwise effects of method on interregional partial correlation.

	TalFr	TalFox	gICA	indICAs
TalFr	—	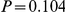	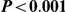	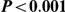
TalFox	n.s.	—	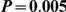	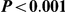
gICA	gICA 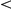 TalFr	gICA 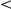 TalFox	—	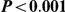
indIAs	indICAs 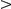 TalFr	indICAs 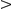 TalFox	indICAs 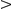 gICA	—

Upper triangular matrix: significance level of a pairwise effect of method. Significant 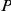-values at a threshold of 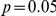 corrected are emphasized in bold. Lower triangular matrix: direction of effect; n.s.: nonsignificant.

#### Global structure

We finally dealt with the whole correlation matrix in order to provide some insight into the global structure of functional connectivity. Using 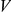 of Equation (6), we quantified the influence of method on within- and between-group variability.

#### Global structure: within-group variability

We first used 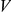 to investigate the effect of method and condition on group variability regarding functional connectivity. We considered 8 groups, each being composed of 14 subjects in either rest or task, and with any of the 4 methods. For each method 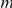 and condition 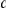, we computed the average correlation matrix characteristic of that method and condition, 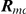, as
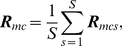
(7)and corresponding measure of group variability:
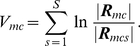
(8)If the group were very homogeneous, then all correlation matrices would be similar and, consequently, 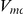 would have a low value. By contrast, if the subjects had very different correlation matrices, then the corresponding 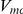 would be large. The results of this analysis are summarized in Table 6, first two rows. Group variability was larger at rest than during the working memory task regardless of method. It was also found to be smaller with gICA than with TalFr or TalFox, and smaller with TalFr or TalFox than with indICAs, regardless of condition.

**Table 6:** Inhomogeneity as a measure of intraclass and interclass variability.

	TalFr	TalFox	gICA	indICAs
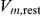	19.0 (51.0%)	19.4 (51.2%)	17.5 (50.6%)	21.2 (48.4%)
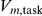	16.3 (43.7%)	16.7 (44.1%)	15.2 (43.9%)	18.1 (41.4%)
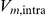	35.2 (94.7%)	36.2 (95.3%)	32.8 (94.5%)	39.3 (89.8%)
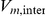	2.0 (5.3%)	1.8 (4.7%)	1.9 (5.5%)	4.4 (10.2%)
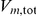	37.2 (100%)	37.9 (100%)	34.7 (100%)	43.7 (100%)
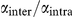	0.056	0.045	0.058	0.113

All quantities are defined in the text, see Equations (8)–(13).

#### Global structure: within- versus between-group variability

To obtain a more precise sense of the relative effect induced by method compared to condition, we also set
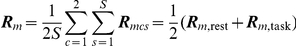
the average correlation matrix corresponding to method 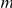, regardless of condition (i.e., the grand average). Similarly to the within- and between-group variance decomposition [51], we defined within-group variability for either the rest or the task condition as in Equation (8), between-group variability as
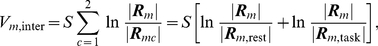
(9)and, finally, total variability as
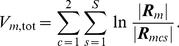
(10)

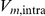
, 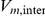, and 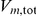 are related by (see Appendix S1)
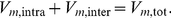
(11)This relationship makes it possible to determine what part of variance is accounted for by group variability
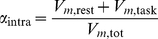
(12)and what part of variance is accounted for by the difference in task,
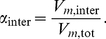
(13)The higher the ratio 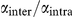, the more we expect to be able to discriminate the effect of the task compared to group variability. The results of the present study are reported in Table 6. Obviously, we found the same results as those presented in the previous section for within-group variability. In terms of percentage, though, intraclass variability increased in the following order: indICAs, gICA, TalFr, and TalFox. Still, in terms of between-group variability, the greatest difference was found using individual ICA, where the ratio 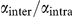 was about twice as large as with the other methods.

#### Multidimensional scaling

**Figure 6:**
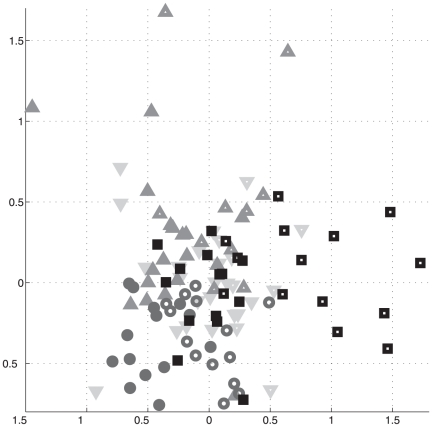
Representation of global MDS. Downward-pointing triangle: TalFr; upward-pointing triangle: TalFox; circle: gICA; square: indICAs. Hallow symbols stand for the rest condition, full ones for the task condition.

**Figure 7:**
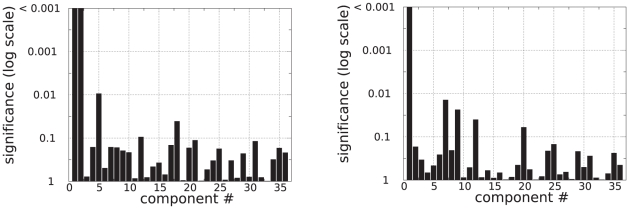
MDS: Component-wise effect of method (left) and condition (right). For each component and corresponding significance level 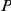, we represented 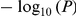.

**Figure 8:**
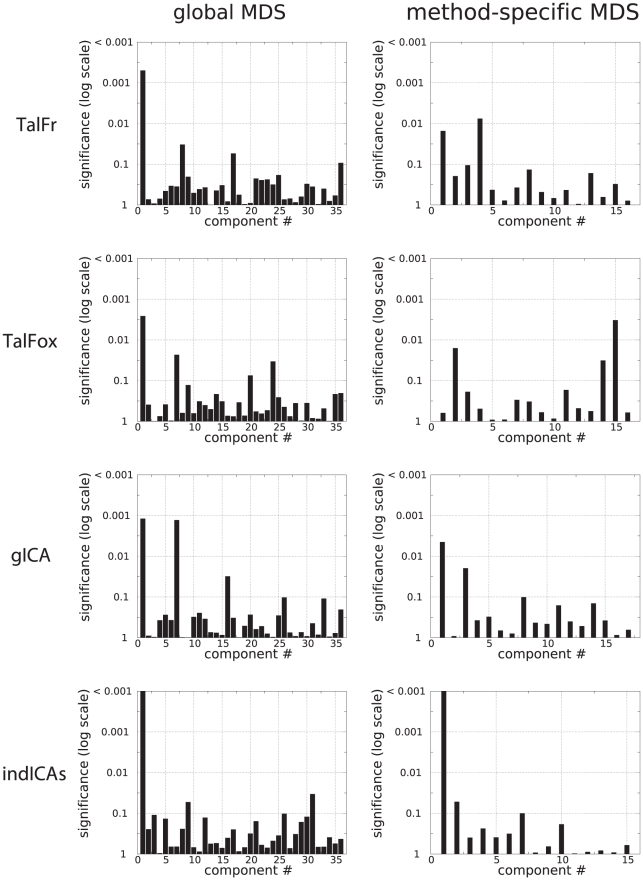
MDS: Effect of condition method by method after MDS on the data from the method only. For each component and corresponding significance level 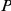, we represented 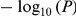.

We finally used 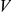 to obtain a global picture of the data. To this aim, we compared any two datasets (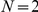) with one another, i.e., for any pair of datasets corresponding to methods 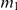 and 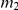, conditions 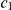 and 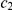, and subjects 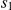 and 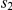,
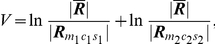
where 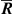 is the average of 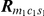 and 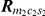. 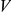 was then used as a distance to perform a 2-dimensional multidimensional scaling (MDS) analysis (procedure implemented in Matlab). 36 components were extracted. A two-dimensional representation of the result is shown in Figure 6. To determine whether MDS was able to summarize the data according to the main effects of interest, we first tested for a global effect of method and condition. We found no significant effect of method (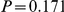) but a significant effect of condition (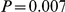). However, neither conclusions were robustly found across conditions or methods, respectively (see Tables 1 and 2). Since MDS components are supposed to provide a classification of information in decreasing order of importance, we also tested for the presence of effects in each component separately. The results are represented in Figure 7. The effect of method was mostly concentrated on the first two components (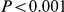 in both cases); no significant effect of method on the other components was found at a threshold of 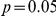 corrected. As to the effect of condition, it was found to be essentially located on the first component (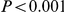). The effect of condition on the first component was rather consistent, since it was observed for three out of four methods (at the exception of TalFox, see Figure 8, left column). The only other component that exhibited a significant effect of condition was component #7 for gICA.

We also performed MDS of the data method by method. In other words, for each method, we only considered data obtained using that method both at rest and during the working memory task condition. The results are summarized in Figure 8, right column. MDS found 16 components for TalFr and TalFox, 17 for gICA, and 15 for indICAs. Only indICAs had a significant effect of condition (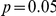 corrected) on a component (component #1). For the three other methods, the effect of condition was spread on various components (e.g., 4 and 1 for TalFr; 15 and 2 for TalFox; 1 and 3 for gICA), leading to subthreshold values of significance.

## Discussion

In the present study, we investigated the influence of four different ROI selection methods in terms of functional connectivity within the DMN. Four different strategies to extract ROIs and corresponding signals were assessed using different approaches. Results are summarized in Table 7 (for method- and condition-specific results, please refer to Tables S1 and S2). First, we compared the ROIs themselves in terms of spatial overlap and within-ROI spatial functional heterogeneity. We found that the spatial overlap between ROIs corresponding to the same region but extracted with different methods was rather limited; often the two volumes were disjoint. We found that the different ROIs had a relatively similar level of spatial functional heterogeneity, even though there was an effect of both method and condition. ROIs extracted with different methods had only limited similarity. We then compared the statistical characteristics of the extracted BOLD signal intensity time courses themselves, in terms of various quantities, such as signal distribution, mean, and variance. Ideally, these quantities should be identical for all ROI selection methods. While this was not the case, we observed that the ROI selection methods had little influence on the marginal distribution of the time series, which we found could be assumed to be normal to a good approximation in most cases. Regarding the characteristic parameters of normal distributions, i.e., mean and variance, we found that, while the ROI selection method had no influence on the signal mean, it had an effect on the signal variance. Regarding condition, we found an effect (a decrease) on signal variance when going from rest to the working memory task. Furthermore, for each of the 9 ROIs selected, all four methods produced correlated signal timecourses and this correlation significantly decreased when going from rest to performing the working memory task. We also assessed the similarities between functional connectivity patterns (as measured by correlation matrices) extracted from different sets of time series. We examined how a change in the exact anatomical location of the ROIs induced changes in the correlation matrix. We showed that the selection method had a global effect on integration , marginal correlation, and partial correlation. Despite these differences, all methods detected a decrease in integration and correlation within the 9 ROIs in the DMN when switching from continuous rest to the continuous verbal 2-back working memory task. We also found that the ROI selection method had a consistent influence on functional connectivity group variability. Still, regardless of that effect, group variability decreased from rest compared to the working memory task. By decomposing total variability into group variability and between-condition variability, we showed that most of the variability was accounted for by group variability; the variability related to condition was small. Nonetheless, MDS made it possible to extract one component for each method that essentially summarized the effect of condition; this component was consistent between methods, because a global MDS showed that only the first component could summarize the condition-induced effect of all methods.

**Table 7:** Summary of results for an effect of method or condition on the different measures of signal or functional connectivity.

		effect of method	effect of condition
ROI features	spatial functional heterogeneity	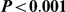	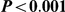
	relative functional heterogeneity	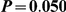	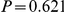
signal features	mean	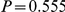	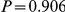
	variance		
	values	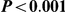	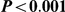
	variations, absolute	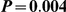	N/A
	variations, relative	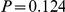	N/A
functional connectivity	integration		
	values	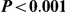	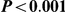
	variations, absolute	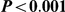	N/A
	variations, relative	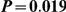	N/A
	marginal correlation		
	values	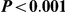	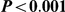
	variations, absolute	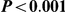	N/A
	variations, relative	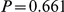	N/A
	partial correlation		
	values	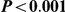	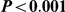
	variations, absolute	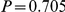	N/A
	variations, relative	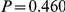	N/A
	MDS	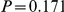	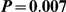

Significant 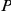-values at a threshold of 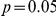 corrected are emphasized in bold. N/A: not applicable.

Altogether, TalFr and TalFox ROI selection procedures produced similar results (in terms of signal variance, network integration, interregional correlation, interregional partial correlation, and inhomogeneity), but differences were nonetheless observed (e.g., in terms of MDS). By contrast, indICAs led to measures of functional connectivity (signal variance, network integration, interregional correlation and partial correlation, between-task inhomogeneity) that were larger than for the three other methods. While different from indICA, results obtained from gICA could not easily be compared with those from TalFr or TalFox. As expected, ROI selection at the individual level, i.e., the indICAs approach, globally yielded a magnification of all estimated differences, be it in terms of measures of interest (e.g., variance, integration) or nuisance factors, such as group variability. IndICAs, which was the most specific to the data in our analysis, yielded the largest changes in terms of the influence of condition; the strongest effects related to the working memory task were often obtained with this method. Nonetheless, while the quantitative results in terms of functional connectivity obtained with the indICA were to some extent different compared to the other methods, we could show that the overall conclusions from the indICA were consistent with those obtained with the other three ROI selection methods.

While the interpretation of the functional connectivity results is not the main focus of the present study, it should still be emphasized that they are in line with our previous study based on the same data [19]. In that study, where we used the indICAs ROI selection method only, we found a global decrease of marginal correlation and a decrease of partial correlation that was limited to a few pairs of regions. In the present study, the global decrease was confirmed with marginal correlation and partial correlation as well as integration with all four ROI selection methods.

It should be noted that we have studied in this investigation the effect of anatomical variability of ROI location and its impact on resting-state functional connectivity. A related question of interest is the potential influence of ROI size and shape on measures of functional connectivity. Given the degree of spatial filtering used and the size of the anatomical regions of interest in the present study, we believe that the spherical ROIs with a radius of 6 mm used here represent a reasonable trade-off between anatomical specificity and signal sensitivity. Regarding size, while selecting spherical ROIs is the most common procedure, other, more refined methods could be used to extract ROIs from the data according to different criteria, such as intra-regional homogeneity [52] or interregional connectivity profiles [53], [54]. A detailed investigation of the relationship between ROI size and shape and resting-state functional connectivity is beyond the scope of the present study.

Although our investigation showed that our main conclusion (a condition-dependent decrease of functional connectivity in the DMN) holds across all ROI selection methods examined, one should bear in mind that all ROI selection methods considered here were based on resting-state data only. Whether this should be considered as a bias or as the cognitive consequence of how the DMN is defined is an issue that remains to be solved. From a methodological perspective, ROI extraction was guided by the neuronal activity that occurred during resting-state conditions only; resulting ROIS were therefore likely to be optimal (in terms of sensitivity) for resting-state conditions but potentially sub-optimal for the working memory task. This fact could potentially introduce a user-derived bias for our finding that explains why we found that all measures of functional connectivity within the DMN were lower during the working memory task than during rest. In that perspective, ruling out the existence of a method-induced, hypothesis-unspecific decrease of functional connectivity should be a matter of concern, which could be solved, e.g., by finding a specific, hypothesis-driven increase of functional connectivity within the same regions. However, it is important to bear in mind that, from a cognitive perspective, the concept of DMN was based on resting-state PET and fMRI data [55], [56]. It therefore seems natural that the definition of ROIs within the DMN should be guided by resting-state data. In more general terms, we believe that the decision of which task condition to use for ROI based sampling of functional connectivity during steady-state conditions should be made with a consideration of which cognitive hypothesis one wishes to test.

As a side remark, we found values of functional heterogeneity that were always larger than one. This means that the time series of all voxels within a given ROI could not be considered as identical, up to some noise. This result provides evidence against the usual representation of a ROI by one time series only, since doing so seems to entail information loss of some sort. Whether this lost information is relevant for connectivity analysis is an issue that remains to be investigated.

In this paper, we sought to answer the question “Which ROI selection technique should be used in the analysis of resting-state functional connectivity?” by a detailed comparison of different strategies and their potential impact on connectivity measures. As expected, we found that the method individualizing the placement of the ROIs provided the best results. By “best”, we here mean that the method yielded results that showed the greatest difference between the rest and the working memory tasks in terms of both the functional connectivity measures (integration and marginal correlation) and the part of variance that could be accounted for by the task (as opposed to between-subject variability). Overall, however, our results support the notion that a moderate variability in anatomical location has a rather limited impact on resting-state functional connectivity within the DMN. Although differences in integration and marginal correlation were detected, all ROI selection schemes reliably detected decreases in connectivity within the DMN for the rest to a working memory transition. While it is often optimal to perform individual ROI selection, our result hints that using group ROIs instead may not lead to a significant loss of information. This result could prove useful in cases where individual ROI selection cannot be performed, e.g., when considering small groups of subjects or individual patients. Note also that non-individual ROI selection methods have the advantage of not making use of the same data twice—first for ROI selection, then for functional connectivity analysis—, a procedure that could be critized from the point of view of frequentist statistics. Moreover, our finding that group variability was larger than variability between tasks warrants some caution to be exerted when comparing functional connectivity between cohorts of patient populations or between different mental states.

Finally, the present study relies on the assumption that there is a change in functional connectivity induced by the change in condition from rest to task, and that this change was fully captured by the correlation matrix. We proved that, under such assumption, the four tested ROI selection methods provided similar conclusions in terms of functional connectivity within the DMN.

## Supporting Information

Figure S1 **Detailed spatial overlaps between ROIs** between TalFr and TalFox (circle), TalFr and gICA (square), and TalFox and gICA (diamond). If *S*_1_ and *S*_2_ are the spheres extracted for a given ROI by methods 1 and 2, respectively, then the overlap between methods 1 and 2 for that ROI is computed as volume(*S*_1_ ∩ *S*_2_)/{[volume(*S*_1_)+volume (*S*_2_)]/2}.
(0.01 MB EPS)

Figure S2 **Detailed spatial overlaps between ROIs** between indICAs and the three other methods. If *S*_1_ and *S*_2_ are the spheres extracted for a given ROI by methods 1 and 2, respectively, then the overlap between methods 1 and 2 for that ROI is computed as volume(*S*_1_ ∩ *S*_2_)/{[volume(*S*_1_)+volume (*S*_2_)]/2}. The bottom and top of the box are the 25th and 75th percentile (the lower and upper quartiles, respectively), and the band in the box is the 50th percentile (median); whiskers represent minimum and maximum values.
(0.08 MB PDF)

Figure S3 **Detailed distances between ROI centers** between TalFr and TalFox (circle), TalFr and gICA (square), and TalFox and gICA (diamond).
(0.01 MB EPS)

Figure S4 **Detailed distances between ROI centers** as extracted with indICAs and the three other methods. The bottom and top of the box are the 25th and 75th percentile (the lower and upper quartiles, respectively), and the band in the box is the 50th percentile (median); whiskers represent minimum and maximum values.
(0.10 MB PDF)

Table S1 **Method-specific effect of condition.** Method-by-method *P*-values for an effect of condition. MDS is performed on the components obtained for a given method after MDS on all the data. MDS^*^ is performed on the components obtained for a given method after MDS on the data corresponding to that method only.
(0.01 MB PDF)

Table S2 **Condition-specific effect of method.** Condition-by-condition *P*-values for an effect of method. MDS is performed on the components obtained for a given method after MDS on all the data. MDS^*^ is performed on the components obtained for a given method after MDS on the data corresponding to that method only.
(0.01 MB PDF)

Appendix S1 Proof of Equation (11).
(0.07 MB PDF)
