# Supplementary figures and images for: Assessing the Influence of Different ROI Selection Strategies on Functional Connectivity Analyses of fMRI Data Acquired During Steady-State Conditions
Source: PLoS One. 2011 Apr 13;6(4):e14788. doi: 10.1371/journal.pone.0014788 (PMC3076321; doi:10.1371/journal.pone.0014788)

### between TalFr and indICAs

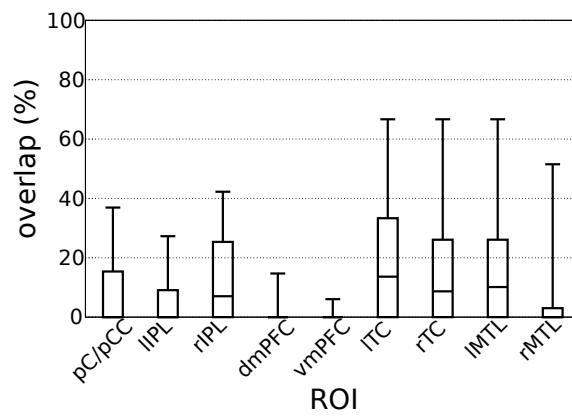

### between TalFox and indICAs

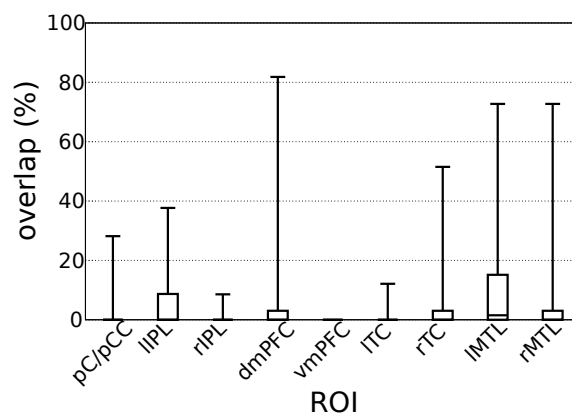

### between gICA and indICAs

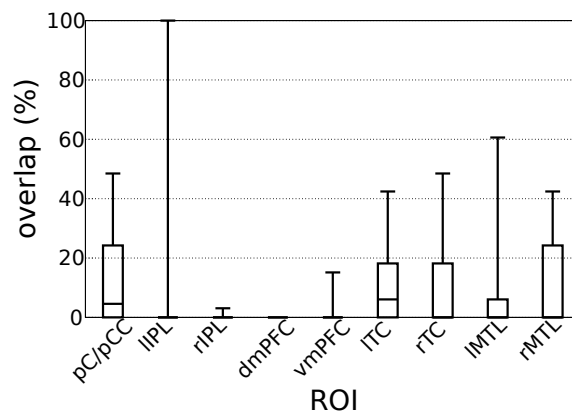

Supplement: Figure S2 — Detailed spatial overlaps between ROIs between indICAs and the three other methods. If S 1 and S 2 are the spheres extracted for a given ROI by methods 1 and 2, respectively, then the overlap between methods 1 and 2 for that ROI is computed as volume(S 1 ∩ S 2)/{[volume(S 1)+volume (S 2)]/2}. The bottom and top of the box are the 25th and 75th percentile (the lower and upper quartiles, respectively), and the band in the box is the 50th percentile (median); whiskers represent minimum and maximum values. (0.08 MB PDF) [file pone.0014788.s002.pdf]

### between TalFr and indICAs

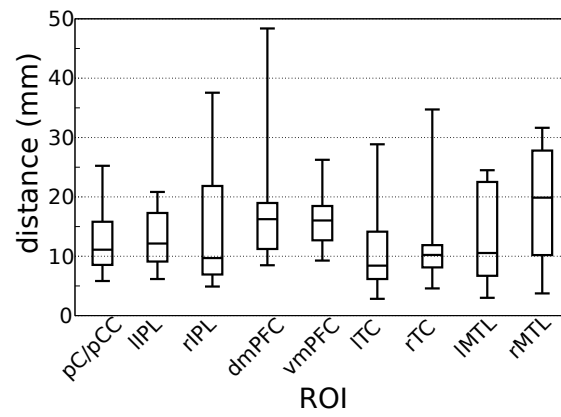

### between TalFox and indICAs

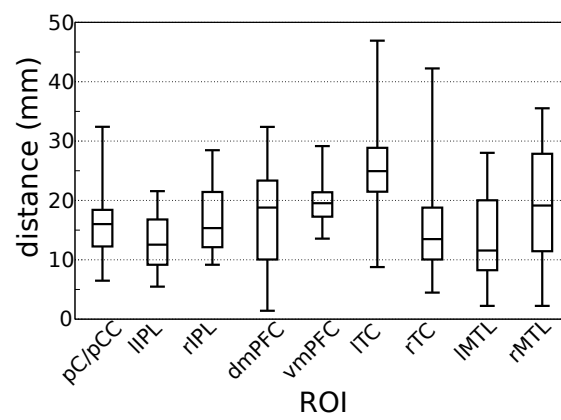

### between gICA and indICAs

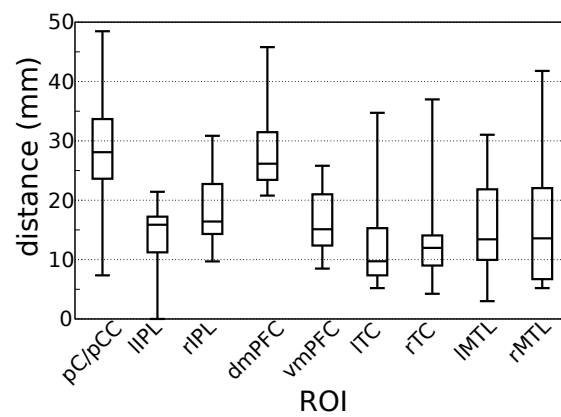

Supplement: Figure S4 — Detailed distances between ROI centers as extracted with indICAs and the three other methods. The bottom and top of the box are the 25th and 75th percentile (the lower and upper quartiles, respectively), and the band in the box is the 50th percentile (median); whiskers represent minimum and maximum values. (0.10 MB PDF) [file pone.0014788.s004.pdf]
