# Supplementary material for: Assessing the Influence of Different ROI Selection Strategies on Functional Connectivity Analyses of fMRI Data Acquired During Steady-State Conditions
Source: PLoS One. 2011 Apr 13;6(4):e14788. doi: 10.1371/journal.pone.0014788 (PMC3076321; doi:10.1371/journal.pone.0014788)
Supplement: Table S1 — Method-specific effect of condition. Method-by-method P-values for an effect of condition. MDS is performed on the components obtained for a given method after MDS on all the data. MDS* is performed on the components obtained for a given method after MDS on the data corresponding to that method only. (0.01 MB PDF) [file pone.0014788.s005.pdf]

	TalFr	TalFox	gICA	indICAs
spatial functional heterogeneity	< 0.001	< 0.001	< 0.001	< 0.001
mean	0.516	0.797	0.612	0.927
variance	< 0.001	< 0.001	< 0.001	< 0.001
integration	0.002	0.060	0.004	< 0.001
marginal correlation	< 0.001	0.016	< 0.001	< 0.001
partial correlation	0.001	0.001	< 0.001	< 0.001
MDS	0.106	0.506	0.839	0.022
MDS*	0.255	0.378	0.115	0.007
