# Supplementary material for: Assessing the Influence of Different ROI Selection Strategies on Functional Connectivity Analyses of fMRI Data Acquired During Steady-State Conditions
Source: PLoS One. 2011 Apr 13;6(4):e14788. doi: 10.1371/journal.pone.0014788 (PMC3076321; doi:10.1371/journal.pone.0014788)
Supplement: Table S2 — Condition-specific effect of method. Condition-by-condition P-values for an effect of method. MDS is performed on the components obtained for a given method after MDS on all the data. MDS* is performed on the components obtained for a given method after MDS on the data corresponding to that method only. (0.01 MB PDF) [file pone.0014788.s006.pdf]

	rest	task
spatial functional heterogeneity	< 0.001	< 0.001
mean	0.437	0.848
variance	< 0.001	< 0.001
integration	< 0.001	0.022
marginal correlation	< 0.001	0.003
partial correlation	< 0.001	< 0.001
MDS	0.015	0.957
MDS*	0.001	0.280
