# Supplementary material for: Assessing the Influence of Different ROI Selection Strategies on Functional Connectivity Analyses of fMRI Data Acquired During Steady-State Conditions
Source: PLoS One. 2011 Apr 13;6(4):e14788. doi: 10.1371/journal.pone.0014788 (PMC3076321; doi:10.1371/journal.pone.0014788)
Supplement: Appendix S1 — Proof of Equation (11). (0.07 MB PDF) [file pone.0014788.s007.pdf]

Appendix S1. Proof of Equation (11)

$$
\begin{aligned}
M_{m,\text{intra}} + M_{m,\text{inter}} &= \sum_{t=1}^{2}\sum_{s=1}^{S} \ln \frac{|\boldsymbol{R}_{mt}|}{|\boldsymbol{R}_{mts}|} + S\sum_{t=1}^{2} \ln \frac{|\boldsymbol{R}_{m}|}{|\boldsymbol{R}_{mt}|} \\
&= \sum_{t=1}^{2}\sum_{s=1}^{S} \left[\ln \frac{|\boldsymbol{R}_{mt}|}{|\boldsymbol{R}_{mts}|} + \ln \frac{|\boldsymbol{R}_{m}|}{|\boldsymbol{R}_{mt}|} \right] \\
&= \sum_{t=1}^{2}\sum_{s=1}^{S} \ln \frac{|\boldsymbol{R}_{m}|}{|\boldsymbol{R}_{mts}|} \\
&= M_{m,\text{tot}}.
\end{aligned}
$$
